# The prognostic value of preoperative serum lactate dehydrogenase levels in patients underwent curative‐intent hepatectomy for colorectal liver metastases: A two‐center cohort study

**DOI:** 10.1002/cam4.4315

**Published:** 2021-10-12

**Authors:** Long Bai, Ze‐Yu Lin, Yun‐Xin Lu, Qin Chen, Han Zhou, Qi Meng, Chun‐Ping Lin, Wan‐Lan Huang, Yun‐Le Wan, Zhi‐Zhong Pan, De‐Shen Wang

**Affiliations:** ^1^ State Key Laboratory of Oncology in South China Collaborative Innovation Center for Cancer Medicine Sun Yat‐sen University Cancer Center Sun Yat‐sen University Guangzhou China; ^2^ Research Unit of Precision Diagnosis and Treatment for Gastrointestinal Cancer Chinese Academy of Medical Sciences Guangzhou China; ^3^ Department of VIP region Sun Yat‐Sen University Cancer Center Guangzhou China; ^4^ Department of Hepatobiliary Surgery The Sixth Affiliated Hospital Sun Yat‐sen University Guangzhou China; ^5^ Department of Medical Oncology Sun Yat‐Sen University Cancer Center Guangzhou China; ^6^ Department of Medical Administration The Sixth Affiliated Hospital Sun Yat‐sen University Guangzhou China; ^7^ Department of Oncology Jieyang Affiliated Hospital Sun Yat‐sen University Jieyang China; ^8^ Department of Colorectal Surgery Sun Yat‐Sen University Cancer Center Guangzhou China

**Keywords:** circulating biomarker, colorectal liver metastases (CRLM), hepatectomy, lactate dehydrogenase (LDH)

## Abstract

**Background:**

The prognostic value of lactate dehydrogenase (LDH) in colorectal cancer patients has remained inconsistent between nonmetastatic and metastatic settings. So far, very few studies have included LDH in the prognostic analysis of curative‐intent surgery for colorectal liver metastases (CRLM).

**Patients and Methods:**

Five hundred and eighty consecutive metastatic colorectal cancer patients who underwent curative‐intent CRLM resection from Sun Yat‐sen University Cancer Center (434 patients) and Sun Yat‐sen University Sixth Affiliated Hospital (146 patients) in 2000–2019 were retrospectively collected. Overall survival (OS) was the primary end point. Cox regression model was performed to identify the prognostic values of preoperative serum LDH levels and other clinicopathology variables. A modification of the established Fong CRS scoring system comprising LDH was developed within this Chinese population.

**Results:**

At the median follow‐up time of 60.5 months, median OS was 59.5 months in the pooled cohort. In the multivariate analysis, preoperative LDH >upper limit of normal (250 U/L) was the strongest independent prognostic factor for OS (HR 1.73, 95% confidence interval [CI], 1.22–2.44; *p* < 0.001). Patients with elevated LDH levels showed impaired OS than patients with normal LDH levels (27.6 months vs. 68.8 months). Five‐year survival rates were 53.7% and 22.5% in the LDH‐normal group and LDH‐high group, respectively. Similar results were also confirmed in each cohort. In the subgroup analysis, LDH could distinguish the survival regardless of most established prognostic factors (number and size of CRLM, surgical margin, extrahepatic metastases, CEA, and CA19‐9 levels, etc.). Integrating LDH into the Fong score contributed to an improvement in the predictive value.

**Conclusion:**

Our study implicates serum LDH as a reliable and independent laboratory biomarker to predict the clinical outcome of curative‐intent surgery for CRLM. Composite of LDH and Fong score is a potential stratification tool for CRLM resection. Prospective, international studies are needed to validate these results across diverse populations.

## Graphical abstract

We retrospectively analyzed 580 patients with colorectal liver metastases (CRLM) who underwent curative‐intent hepatectomy. Our study implicated preoperative LDH level as a reliable and independent laboratory biomarker to predict the clinical outcome of curative‐intent surgery for CRLM. Composite of LDH and Fong score is a potential stratification tool for CRLM resection.

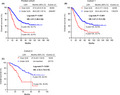

## 1 INTRODUCTION

Colorectal cancer (CRC) is the third most common cancer and the second leading cause of cancer‐related mortality worldwide.^1^, ^2^, ^3^ The liver is the primary life‐limiting distant metastatic site for CRC.^4^ About a quarter of CRC patients present concurrent liver metastases, and over half will develop liver metastases through the course of diseases.^5^ Surgical excision‐based locoregional therapy remains the only possible curative option for colorectal liver metastases (CRLM).^6^ However, only about 20% of CRLM patients are candidates for curatively intended liver resection at diagnosis.^7^ Whereas a growing number of curative hepatectomy has been achieved through multidisciplinary therapy within the latest decade, most patients (50%–80%) would develop a further recurrence.^8^, ^9^ The survival outcomes derived from different studies remain heterogeneous, with 5‐year survival rates ranging from 25% to 60%.^6^, ^10^, ^11^ Thus, a better selection of patients before initiating treatment is needed to refine the therapeutic decisions.

Recent studies have shown that apart from conventional clinicopathology variables, gene expression signatures, intratumoral immune cell infiltrations, and circulating tumor cells also have a prognostic impact on colorectal cancer.^10^, ^11^, ^12^ In particular, the serum biochemical markers, namely Gamma‐glutamyl transferase (GGT), alkaline phosphatase (ALP), and lactate dehydrogenase (LDH), also gained the appreciation for their prognostic implications in mCRC.^13^, ^14^

As the key enzyme in aerobic and anaerobic glycolysis, LDH plays a pivotal role in tumor metabolism by mediating the conversion of pyruvate and lactate.^15^ Evidence is emerging that LDH is closely related to hypoxia, angiogenesis, inflammation, and immune status in the tumor microenvironment (TME). High serum LDH levels indicate poor prognosis among various cancer entities and promote resistance to chemo/radio/targeted therapy.^16^, ^17^, ^18^ However, the prognostic value of LDH in CRC has remained inconsistent between nonmetastatic and metastatic settings.^19^, ^20^, ^21^ Elevated circulating LDH levels were reported to be an adverse prognostic factor in unresectable CRLM patients receiving systemic therapy or hepatic arterial infusion.^22^, ^23^, ^24^ In contrast, this effect was not evident for the overall survival of nonmetastatic CRC patients.^25^, ^26^ Moreover, very few studies have included LDH in the prognostic analysis of curative‐intent surgery for CRLM. Therefore, it remains to be determined whether preoperative LDH levels could predict the outcome of complete CRLM resection, in which situation patients usually achieve a no evidence of disease (NED) status.

To address this issue, we performed this two‐center, retrospective observational study in a cohort of 580 patients with resected CRLM. Our objectives are (a) to evaluate the prognostic impact of preoperative serum LDH levels on curative‐intent surgery for CRLM and (b) to integrate LDH into the established Fong scoring system within this Chinese population to improve patient stratification for CRLM resection.

## 2 MATERIALS AND METHODS

### 2.1 Study population

This two‐center, retrospective cohort study enrolled consecutive 580 histologically proven CRLM patients who underwent curative‐intent hepatectomy in Sun Yat‐sen University Cancer Center (cohort 1) and Sun Yat‐sen University Sixth Affiliated Hospital (cohort 2). Cohort 1 included 434 patients from September 2000 to December 2016, while cohort 2 included 146 patients from August 2012 to June 2019. Detailed clinical information (preoperation and postoperation clinicopathological data, blood examination, follow‐up information, etc.) was retrieved from electronic‐ and paper‐based medical records from each center.

The inclusion criteria were listed as follows: (1) histologically confirmed colorectal adenocarcinoma, (2) patients who underwent curative‐intent CRLM resection, (3) postoperative follow‐up period of at least 3 months, and (4) preoperative serum LDH values had to be available within 2 weeks before hepatectomy.

The exclusion criteria were listed as follows: (1) peritoneal metastasis, (2) previous history of hepatectomy, (3) R2 resection of liver metastases, (4) ablation of metastatic sites or transcatheter hepatic arterial chemoembolization (TACE) within 4 weeks of study entry, (5) patients in inflammatory conditions, and (6) previous history of malignant tumor.

This was a noninterventional, observational, and retrospective study in which the patient data used were kept strictly confidential. All patients were provided written consent for the use of their data at the time of hospitalization. The study was performed following the Helsinki declarations and the ethics committee from both centers. The originality and authenticity of this article have been validated by uploading the key raw data onto the Research Data Deposit public platform (www.researchdata.org.cn).

### 2.2 Follow‐up

Overall survival (OS) was defined as the time from hepatic resection to death from any cause or latest follow‐up. Recurrence‐free survival (RFS) was measured from the date of hepatic resection to confirming recurrence or death for any reason, whichever occurred first. Patients were followed up through outpatient clinical visits or via telephone. The follow‐up starts 1 month after the operation and ends when tumor relapse or death was verified, while subjects who were lost or still alive at the date of the last contact were considered censored.

### 2.3 Blood sample test

Data from blood examination (blood routine tests, blood chemistry tests, and tumor marker tests) were eligible for analysis if performed within 2 weeks before hepatectomy. The blood examination was performed by each center's laboratory. Enrolled patients were divided into LDH‐normal and LDH‐high groups, using the upper limit of normal (ULN) established by each center's laboratory as the cutoff value, in anticipation of elaborating a practical clinical tool for future use. The ULN of LDH at both centers was 250 U/L. Preoperative immune/inflammation‑related factors (including neutrophil, lymphocyte, monocyte, and platelet counts, LMR, LNR, and LPR, and C‐reaction protein) were collected. LMR, LNR, and LPR were defined as absolute lymphocyte count divided by absolute monocyte, neutrophil, and platelet count, respectively.

### 2.4 Modified Clinical Risk Score establishment and validation

The clinical risk score (CRS) was calculated according to the criteria initiated by Yuman Fong.^27^ Briefly, five clinical criteria, primary lymph node‐positive, the disease‐free interval from the diagnosis of primary tumor <12 months, number of CRLM >1, maximum CRLM diameter >5 cm, preoperative CEA levels >200 ng/ml, were assigned one point for each and total scores were defined as CRS. We integrated preoperative LDH levels into the CRS model to test whether the predictive ability improved. Two models were established as follows:

(a) LDH was added to the CRS model (LDH‐CRS). The LDH‐CRS was calculated as follows: primary lymph node‐positive, the disease‐free interval from the diagnosis of primary tumor <12 months, number of CRLM >1, maximum CRLM diameter >5 cm, preoperative CEA levels >200 ng/ml, and preoperative LDH levels >ULN were assigned one point for each and total scores were defined as LDH‐CRS.

(b) Preoperative CEA levels were replaced by LDH levels (modified CRS [mCRS]). The mCRS was calculated as follows: primary lymph node‐positive, the disease‐free interval from the diagnosis of primary tumor <12 months, number of CRLM >1, maximum CRLM diameter >5 cm, and preoperative LDH levels >ULN were assigned one point for each and total scores were defined as mCRS.

The discriminatory ability of models was assessed by area under the curve (AUC) in the time‐dependent receiver operating characteristic (ROC) analysis. Harrell's discrimination concordance index (C‐index, which is defined as the probability that predictions and outcomes are concordant) was employed to validate the predictive ability of the models.

### 2.5 Statistical analysis

Patients’ characteristics between different groups were compared with Student's *t*‐test, χ^2^, Wilcoxon rank‐sum test, or Kruskal–Wallis test as statistically appropriate. The survival curves were generated using the Kaplan–Meier method and compared with the log‐rank test in terms of RFS and OS.

OS was the primary end point. To identify independent prognostic predictors, univariate and multivariate Cox proportional hazard regression analyses were performed. The associations between baseline clinicopathologic variables (age, gender, primary tumor location, grade of differentiation, pathology, T and N stage of primary tumor, preoperative CEA and CA19‐9 levels, LDH level, number of CRLM, maximum diameter of CRLM, extrahepatic metastases, surgical margin of CRLM, preoperative chemotherapy, disease‐free interval from discovery of primary tumor to liver metastases) and survival outcome were explored and quantified by hazard ratios (HRs) and corresponding 95% confidence intervals (CIs). Parameters with *p* < 0.10 in the univariate analysis were selected and further included in the multivariate analysis, relying on the ENTER algorithm with a selected level of 0.05. In the multivariable analysis for pooled population, the cohort was obligated to be an adjustment factor to exclude the confounding factor of different affiliates.

*KRAS* and *BRAF* mutation was not considered for Cox regression analysis because it was not available for all patients, especially for patients in cohort 2 (Table 1). Hence, a sensitivity analysis in cases with available data of *KRAS* mutation status was performed in a multivariable model.

**TABLE 1:** Clinicopathologic characteristics of patients

Variables	Frequencies, *n* (%) (*n* = 580)
Patient characteristics	
Cohort 1	434 (74.8)
Cohort 2	146 (25.2)
Age: median (range)	59 (20–82)
Gender	
Male	385 (66.4)
Female	195 (33.6)
Preoperative CEA	
> 5 ng/ml	349 (60.1)
≤ 5 ng/ml	26 (35.5)
Missing	26 (4.5)
Preoperative LDH	
Over ULN	93 (16.0)
Under ULN	487 (84.0)
Survival outcome	
Median follow‐up (month)	60.5 (95% CI, 57.5–63.5)
Median OS (month)	59.5 (95% CI, 58.4–70.6)
Primary tumor characteristics	
Location^a^	
Right‐sided	139 (24.0)
Left‐sided	340 (58.6)
Missing	101(17.4)
Differentiation	
Well/moderate	459 (79.1)
Poor	121 (20.9)
Pathology	
Adenocarcinoma	543 (93.5)
Signet‐ring cell/mucinous carcinoma	38 (6.5)
Lymph node metastases	
Absent	197 (34.0)
Present	305 (52.6)
Missing	78 (13.4)
CRLM characteristics	
Maximum diameter of CRLM	
≤ 5cm	490 (84.5)
> 5cm	80 (13.8)
Missing	10 (1.7)
Number of CRLM	
1	262 (45.1)
> 1	319 (54.9)
Time of occurrence of CRLM	
Synchronous	440 (75.9)
Metachronous	140 (24.1)
*KRAS*	
Wild type	126 (21.7)
Mutated	52 (9.0)
Missing	(69.3)
Extrahepatic metastases	
Yes	49 (8.4)
No	531 (91.6)
R0 resection	
Yes	504 (86.9)
No	72 (12.4)
Missing	4 (0.7)
Perioperative treatment	
Preoperative chemotherapy alone	167 (28.8)
Preoperative bevacizumab treatment	44 (7.6)
Preoperative cetuximab treatment	41 (7.1)
Postoperative chemotherapy	437 (77.1)

Abbreviations: CRLM, colorectal liver metastases; LDH, lactate dehydrogenase; OS, overall survival; ULN, upper limit of normal.

^a^ Colorectal cancer arising in or proximal to the splenic flexure was defined as right‐sided; arising distal to the splenic flexure was defined as left‐sided.

Furthermore, subgroup analyses were carried out stratified by demographic and clinicopathologic variables and presented by forest plots.

As for the comparison of time‐dependent AUC between different models, the Wilcoxon matched‐pair signed‐rank test was applied. Time‐dependent AUC was calculated by the Package timeROC (version 0.4). The C‐index was calculated by the Package rms (version 5.1–3.1). Statistical analyses were conducted with the SPSS software version 19 (SPSS, Chicago, IL), STATA (Release 14.2; StataCorp LP, College Station, TX), and GraphPad Prism 7.0.

## 3 RESULTS

### 3.1 Characteristics of patients

A total of 580 consecutive CRLM patients at two Chinese medical centers were enrolled. Four hundred and thirty‐four patients were from cohort 1, and 146 patients were from cohort 2. Clinicopathology and treatment characteristics were listed in Table 1. All enrolled patients were Chinese individuals, the average age at diagnosis was 59. The median follow‐up time was 60.5 months, whereas median OS was 59.5 months (95% CI, 58.4–70.6) in the pooled cohort, 58.9 months (95% CI, 46.2–71.6) in cohort 1, and 63.3 months (95% CI, 61.3–67.8) in cohort 2, respectively (Table S1).

Overall, clinical features were well balanced between the two cohorts, except patients in cohort 1 had a higher proportion of synchronous CRLM (95.2% vs. 69.4%), T4 stage of the primary tumor (83.2% vs. 64%), well to moderately differentiated pathology (87.7% vs. 76.3%), and LDH levels above ULN (23.3% vs. 13.6%) than patients in cohort 2 (Table S1).

### 3.2 LDH levels and correlations with clinical characteristics

The relationship between serum LDH and clinicopathologic parameters was detailed in Table 2. In summary, serum LDH levels showed no statistical difference when stratified by demography characteristics (age and gender), primary tumor characteristics (tumor location, pathology differentiation, and T and N stage), metastatic site characteristics (presence of extrahepatic disease, number of CRLM, and perioperative chemotherapy), and *KRAS* and *BRAF* mutations (data not shown in Table 2 because gene test was not carried out in a minority of patients).

**TABLE 2:** Relationship between patient characteristics and LDH levels in the pooled cohort

Variables	LDH levels		*p* value
	Under ULN, *n* (%)	Over ULN, *n* (%)	
Patient characteristics			
Age			0.245
≤59^a^	274 (85.6)	46 (14.4)	
>59	196 (81.7)	44 (18.3)	
Gender			0.281
Male	328 (85.2)	57 (14.8)	
Female	159 (81.5)	36 (18.5)	
Preoperative CEA			0.011*
≤5 ng/ml	184 (89.3)	22 (10.7)	
>5 ng/ml	283 (81.1)	66 (18.9)	
Preoperative CA19‐9			0.009
≤35 U/L	312 (87.2)	46 (12.8)	
>5 U/L	145 (78.4)	40 (21.6)	
Primary tumor characteristics			
Location^b^			0.428
Right‐sided	120 (86.3)	19 (13.7)	
Left‐sided	366 (83.2)	74 (16.8)	
Pathology			0.249
Adenocarcinoma	452 (83.4)	90 (16.6)	
Signet‐ring cell/mucinous carcinoma	35 (92.1)	3 (7.9)	
Differentiation			0.404
Well/moderate	382 (83.2)	77 (16.8)	
Poor	105 (86.8)	16 (13.2)	
T stage			0.449
Non‐T4	320 (84.9)	57 (15.1)	
T4	139 (82.2)	30 (17.8)	
Lymph node metastases			0.275
Absent	180 (86.5)	28 (13.5)	
Present	272 (82.7)	57 (17.3)	
CRLM characteristics			
Maximum diameter of CRLM			<0.001*
≤2.5 cm^c^	278 (90.3)	30 (9.7)	
>2.5 cm	197 (75.8)	63 (24.2)	
Number of CRLM			0.055
1–2^d^	334 (86.1)	64 (13.9)	
>2	153 (79.7)	39 (20.3)	
Time of occurrence of CRLM			0.033*
Synchronous	362 (82.1)	79 (17.9)	
Metachronous	125 (89.9)	14 (10.1)	
Extrahepatic disease			1.00
Absent	446 (84.0)	51 (16.0)	
Present	41 (83.7)	8 (16.3)	
Perioperative chemotherapy			0.467
Yes	309 (87.0)	46 (13.0)	
No	66 (83.5)	13 (16.5)	
CRS			<0.001*
0–1	94 (91.3)	9 (8.7)	
2–3	309 (85.8)	51 (14.2)	
4–5	21 (48.8)	22 (51.2)	

Abbreviations: CRLM, colorectal liver metastases; CRS, Clinical Risk Score; LDH, lactate dehydrogenase; ULN, upper limit of normal.

^a^ Median age of patients in the pooled cohort was 59.

^b^ Colorectal cancer arising in or proximal to the splenic flexure was defined as right‐sided, and arising distal to the splenic flexure was defined as left‐sided.

^c^ Median maximum diameter of CRLM in the pooled cohort was 2.5 cm.

^d^ Median number of CRLM in the pooled cohort was two.

* Statistical significance

However, we observed patients with a maximum diameter of CRLM ≤2.5 cm (the median diameter) had a higher proportion of elevated LDH levels than patients with a maximum diameter of CRLM >median (24.2% vs. 9.7%, *p* < 0.001). Patients with elevated CEA also had a greater possibility of having elevated LDH than those with normal CEA levels (18.9% vs. 10.7%, *p* = 0.011). A similar trend was observed for CA19‐9 levels (*p* = 0.006). Patients with synchronous CRLM had a higher proportion of elevated LDH than those with metachronous CRLM (17.9% vs. 10.1%, *p* = 0.033). In addition, patients with CRS of 4–5 had a higher proportion of elevated LDH than patients with CRS 2–3 or CRS 0–1 (51.2% vs. 14.2% vs. 8.7%; *p* < 0.001).

### 3.3 Cox regression analysis of relapse‐free survival and overall survival

Due to some missing data for the baseline variables (details in Table 1), 490 patients were finally included in the multivariable model. Elevated preoperative LDH levels (defined as LDH >ULN) were found to be the strongest prognostic factor for OS (Table 3).

**TABLE 3:** Univariate and multivariate analyses for predictors of overall survival in the pooled cohort

Variables	Univariate analysis		Multivariate analysis	
	HR (95% CI)	*p* value	HR (95% CI)	*p* value
Age	1.02 (1.006–1.029)	0.002	1.03 (1.01–1.04)	<0.001*
Gender (male)	1.19 (0.92–1.54)	0.192		
Primary tumor location				
Right‐sided vs. left‐sided ^a^	1.17 (0.88–1.54)	0.277		
Poor differentiation	1.28 (0.96–1.70)	0.088	0.99 (0.70–1.38)	1.38
Signet‐ring cell/mucinous carcinoma	1.09 (0.69–1.73)	0.700		
T4 stage	1.30 (1.00–1.68)	0.050	1.26 (0.94–1.69)	0.126
Primary tumor lymph node metastasis	1.78 (1.36–2.33)	<0.001	1.70 (1.27–2.27)	<0.001*
Preoperative CEA levels	1.67 (1.24–2.26)	0.001	1.23 (0.91–1.67)	0.184
Preoperative CA19‐9 levels	1.68 (1.31–2.17)	<0.001	1.47 (1.09–1.98)	0.012*
Metachronous CRLM	0.82 (0.63–1.07)	0.151		
Number of CRLM	1.19 (1.14–1.25)	<0.001	1.13 (1.07–1.20)	<0.001*
Maximum diameter of CRLM	1.09 (1.04–1.13)	<0.001	1.07 (1.01–1.13)	0.027*
Extrahepatic metastases	1.56 (1.05–2.31)	0.026	1.61 (1.03–2.54)	0.039*
Preoperative chemotherapy	1.51 (1.19–1.92)	0.001	1.35 (0.99–1.86)	0.061
R0 resection	0.37 (0.26–0.53)	<0.001	0.56 (0.37–0.84)	0.006*
LDH levels (> ULN)	2.51 (1.88–3.36)	<0.001	1.73 (1.22–2.44)	<0.001*
Cohort 1 vs. cohort 2	0.89 (0.65–1.21)	0.886	1.27 (0.84–1.93)	0.251

Abbreviations: CI, confidence interval;CRLM, colorectal liver metastases; HR, hazard ratio; ULN, upper limit of normal.

^a^ Colorectal cancer arising in or proximal to the splenic flexure was defined as right‐sided and those arising distal to the splenic flexure was defined as left‐sided.

* Statistical significance.

In the univariate analysis, age, pathology differentiation, T stage of the primary tumor, lymph node metastases of the primary tumor, preoperative CEA and CA19‐9 levels, number of CRLM, maximum diameter of CRLM, presence of extrahepatic metastases, preoperative chemotherapy, R0 resection margin, and LDH levels were significant predictors for OS.

After adjusted for the above clinicopathologic parameters, eight factors were ultimately identified as independent prognostic makers for OS in the multivariate analysis: age (HR, 1.03; 95% CI, 1.01–1.04; *p* < 0.001), lymph node metastasis of the primary tumor (HR, 1.70; 95% CI, 1.27–2.27; *p* < 0.001), preoperative CA19‐9 (HR, 1.47; 95% CI, 1.09–1.98; *p* = 0.012), the number of CRLM (HR, 1.13; 95% CI, 1.07–1.20; *p* < 0.001), the maximum diameter of CRLM (HR, 1.07; 95% CI, 1.01–1.13; *p* < 0.001), extrahepatic disease (HR, 1.61; 95% CI, 1.03–2.54; *p* = 0.039), R0 resection margin (HR, 0.56; 95% CI, 0.37–0.84; *p* = 0.006), and elevated preoperative LDH levels (HR, 1.73; 95% CI, 1.22–2.44; *p* < 0.00781).

In the stratified analyses for each cohort, LDH remained its independent prognostic value for OS in the multivariate analysis, both in cohort 1 (HR, 1.77; 95% CI, 1.17–2.69; *p* < 0.001; Table S2) and in cohort 2 (HR, 3.71; 95% CI, 1.75–7.89; *p* = 0.001; Table S3).

In terms of RFS, LDH remained an independent predictor in the multivariate analysis (HR, 1.53; 95% CI, 1.01–2.03; *p* = 0.042), along with lymph node metastases of the primary tumor, number of CRLM, and the maximum diameter of CRLM (Table S4).

Additionally, in the sensitivity analysis in cases with available data of *KRAS* mutation status, only the number and size of CRLM were independent predictors for OS in multivariable models probably due to the limitation of sample size (Table S5).

### 3.4 Survival outcomes according to LDH levels and subgroups analysis

**FIGURE 1:**
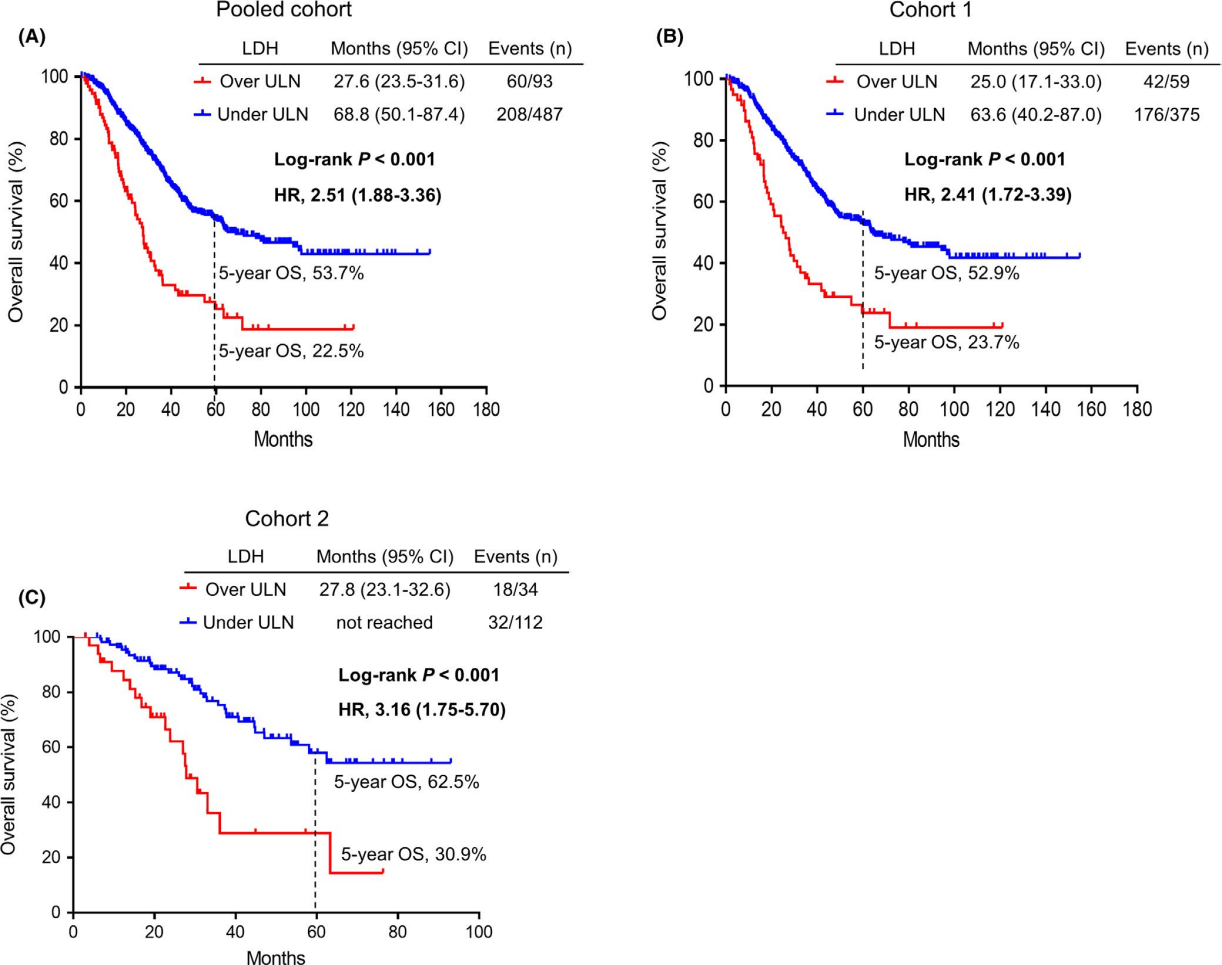
Overall survival according to preoperative serum LDH levels estimated by Kaplan–Meier curves. (A) Overall survival stratified by LDH levels in the pooled cohort. (B) Overall survival stratified by LDH levels in cohort 1. (C) Overall survival stratified by LDH levels in cohort 2. Abbreviations: CI, confidence interval; HR, hazard ratio; LDH, lactate dehydrogenase; ULN, upper limit of normal

In the pooled cohort, patients with elevated LDH showed impaired OS compared with patients with normal LDH levels (27.6 months vs. 68.8 months; HR, 2.51, 95% CI, 1.88–3.36; *p* < 0.001). Survival rates at 5 years in the LDH‐normal and LDH‐high group were 53.7% and 22.5%, respectively. In the stratified analysis, cohort 1 (25.0 months vs. 63.6 months; HR, 2.41, 95% CI, 1.72–3.39; *p* < 0.001) and cohort 2 (27.8 months vs. not reached; HR, 3.16, 95% CI, 1.75–5.70; *p* < 0.001) demonstrated similar results as the pooled cohort (Figure 1).

On the other hand, patients with elevated LDH had significantly shorter RFS (8.5 months vs. 22.0 months; HR, 2.11, 95% CI, 1.54–2.89; *p* < 0.001) than patients with normal LDH levels in cohort 1 (Figure S1).

**FIGURE 2:**
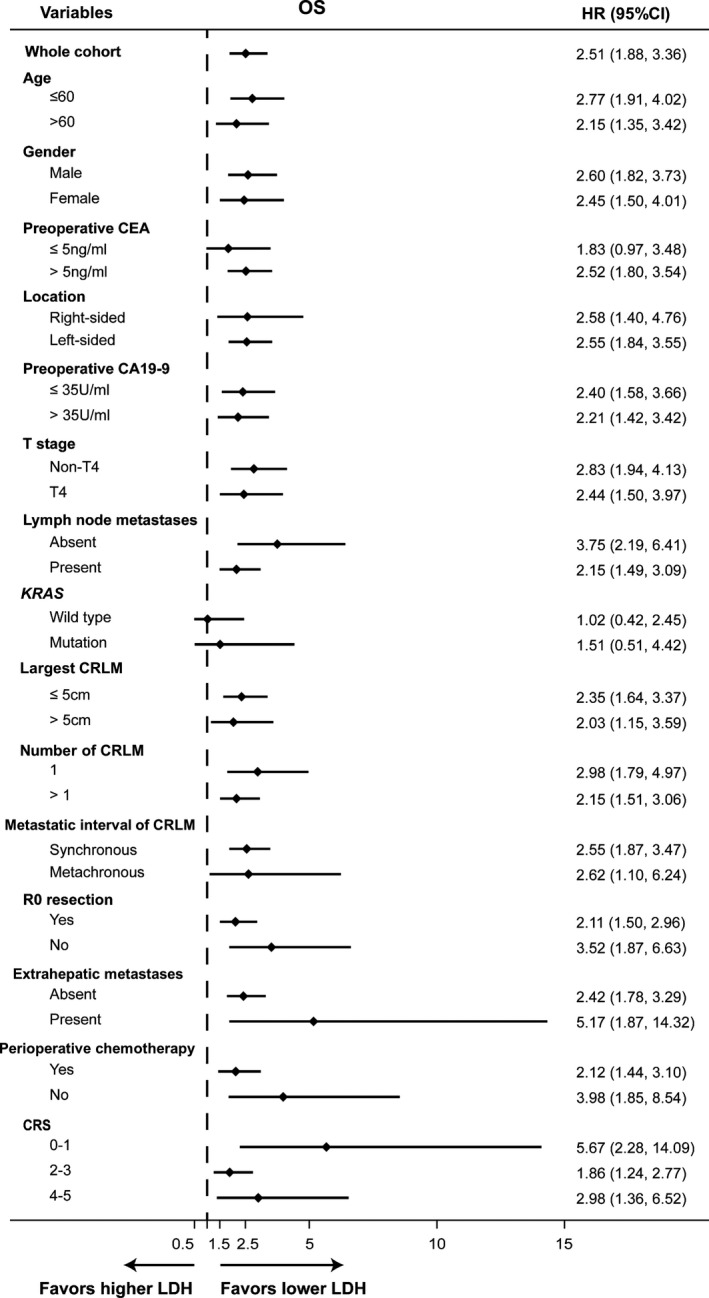
Forest plots of hazard ratios (elevated LDH vs. normal LDH) for overall survival according to subgroups in the pooled cohort. Abbreviations: OS, overall survival; RFS, relapse‐free survival; HR, hazard ratio; CRS, Clinical Risk Score

Subgroup analyses revealed that LDH produced consistent prognostic value across patient subgroups stratified by age, sex, primary tumor characteristics (location, T and N stage), liver metastases characteristics (number, maximum diameter, surgical margin, disease‐free interval from primary tumor, extrahepatic disease), perioperative chemotherapy, preoperative CEA and CA19‐9 levels, even by Fong score. The forest plots provided a clear trend that patients with lower LDH levels obtained better survival benefits from hepatectomy for OS (Figure 2).

### 3.5 Survival outcomes assessed by CRS, LDH‐CRS, and mCRS

**FIGURE 3:**
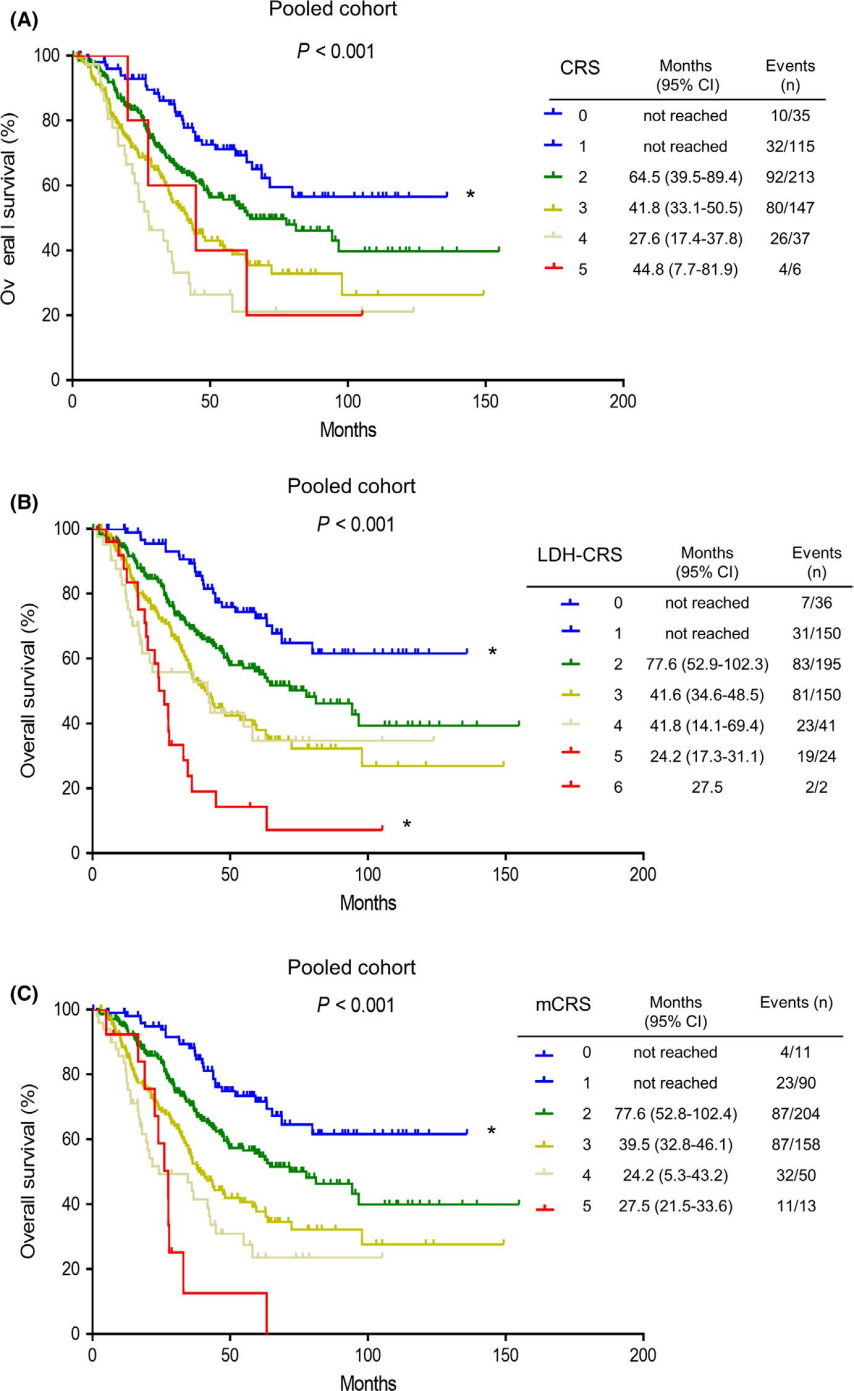
Overall survival stratified by different scoring systems in the pooled cohort. (A) Overall survival stratified by risk scores (0–5) as defined by CRS. (B) Overall survival stratified by risk scores (0–6) as defined by LDH‐CRS. (C) Overall survival stratified by risk scores (0–5) as defined by modified CRS. *Due to the small sample size, scores 0 and 1 were grouped together in all scoring systems; scores 5 and 6 were grouped together in the CRS‐LDH model. Abbreviations: CRS, Clinical Risk Score; mCRS, modified Clinical Risk Score

OS stratified by different risk scores as defined by CRS and LDH‐CRS were demonstrated by Kaplan–Meier curves in the pooled cohort. Median OS of the risk scores 0–5 in the CRS model was not reached, not reached, 64.5 months, 41.8 months, 27.6 months, 44.8 months, respectively. Median OS of risk scores 0–6 in the LDH‐CRS model were not reached, not reached, 77.6 months, 41.6 months, 41.8 months, 24.2 months, 27.5 months, respectively. While median OS of the risk scores 0–5 in the mCRS model were not reached, not reached, 77.6 months, 39.5 months, 24.2 months, 27.5 months, respectively. Particularly, LDH‐CRS and mCRS identified a relatively higher proportion of patients in the high‐risk group (score of 4–6) than CRS (13.2% [67/506] vs. 12.0% [63/526] vs. 8.5% [43/506]) (Figure 3).

On the other hand, LDH levels could overcome the CRS scoring system. 8.7% of the patients in the CRS 0–1 group had LDH >ULN and presented with significantly poor outcomes than patients who had LDH ≤ULN (mOS 29.7 months vs. not reached, *p* = 0.005); conversely, 48.8% of the patients in the CRS 4–5 group had LDH ≤ULN and presented with significantly good outcomes than patients who had LDH >ULN (mOS 44.8 months vs. 24.2 months, *p* < 0.001). In the CRS 2–3 group, 14.2% of patients who had LDH >ULH demonstrated significantly worse OS than patients with LDH ≤ULN (30.5 months vs. 60.2 months, *p* = 0.002) (Figure S3).

### 3.6 Receiver‐operating characteristic (ROC) analysis for the comparison of CRS and LDH‐CRS in prediction ability

**FIGURE 4:**
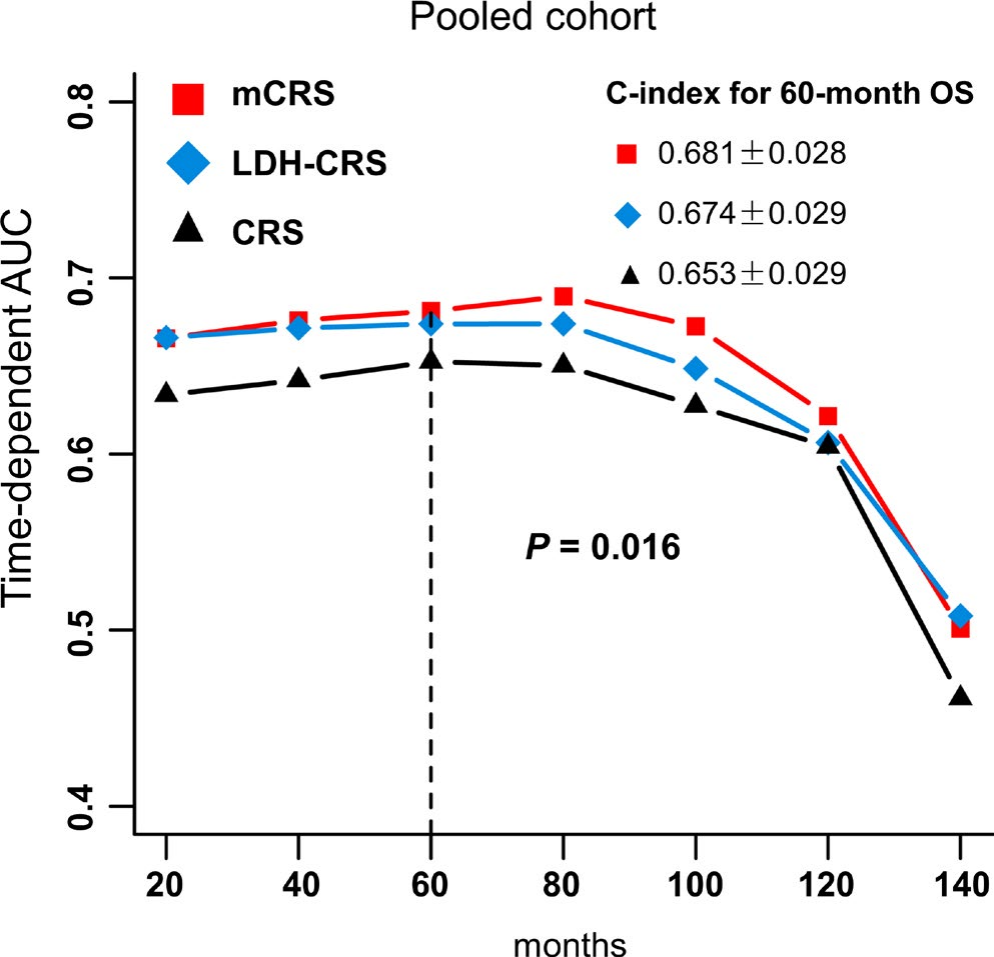
Receiver‐operating characteristic (ROC) analysis for the comparison of different scoring systems in prediction of overall survival in the pooled cohort. Abbreviations: CRS, Clinical Risk Score; mCRS, modified Clinical Risk Score; AUC, area under curve; C‐index, concordance index; OS, overall survival

Time‐dependent ROC analysis displayed that LDH‐CRS and mCRS exhibited a better predictive value than CRS in the pooled cohort for OS (*p* = 0.016). In the CRS model, the C‐index of the 5‐year OS probability forecast was 0.653 ± 0.029, the C‐index of the LDH‐CRS model was 0.674 ± 0.029, whereas the C‐index of the mCRS model was 0.681 ± 0.028 (Figure 4). These results suggest that adding LDH to the CRS scoring system demonstrated a better accuracy.

### 3.7 Association of LDH levels and immune/inflammation‑related indices

**FIGURE 5:**
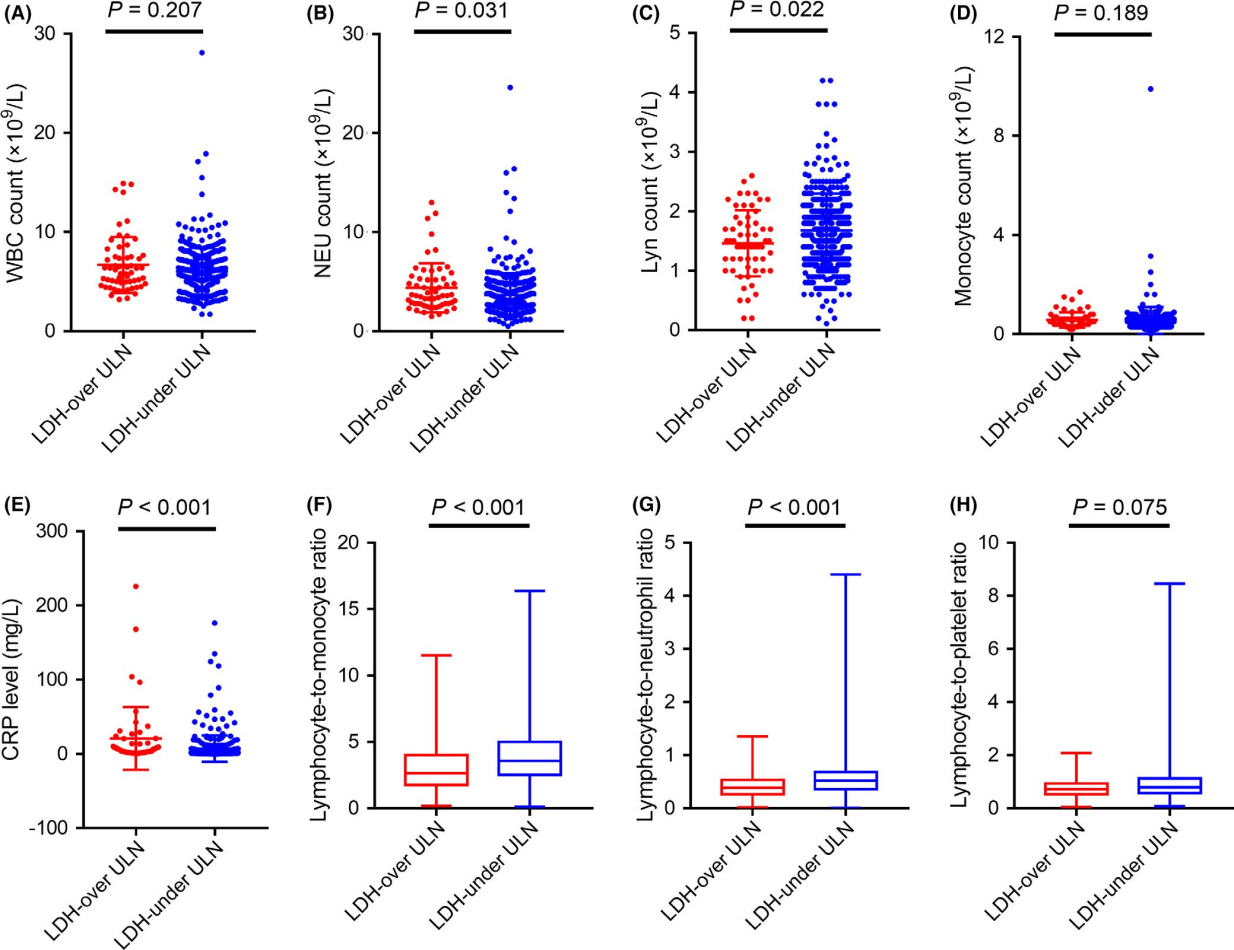
Associations between preoperative serum LDH levels and serum immune/inflammation‐related factors. (A) LDH levels and WBC counts, (B) LDH levels and neutrophil counts, (C) LDH levels and lymphocyte counts, (D) LDH levels and monocyte counts, (E) LDH levels and C‐reactive protein levels, (F) LDH levels and lymphocyte‐to‐monocyte ratios, (G) LDH levels and lymphocyte‐to‐neutrophil ratios, (H) LDH levels and lymphocyte‐to‐platelet ratios. Abbreviations: WBC, white blood cell; NEU, neutrophil; Lyn, lymphocyte; CRP, C‐reactive protein

In an exploratory analysis, it is interesting to note that LDH levels varied with a set of immune/inflammatory factors (Figure 5). Specifically, patients with elevated LDH had higher preoperative neutrophil counts (*p* = 0.031), higher C‐reaction protein (CRP) levels (*p* < 0.001), and lower lymphocyte counts (*p* = 0.022) than patients with normal LDH levels. Consequently, patients with elevated LDH also had a lower lymphocyte‐to‐monocyte ratio (LMR; *p* < 0.001) and lymphocyte‐to‐neutrophil ratio (LNR; *p* < 0.001). On the contrary, LDH levels were not associated with preoperative total white blood cell counts or monocyte counts.

## 4 DISCUSSION

Resection of colorectal liver metastases is fraught with high rates of recurrence. It represents an area of intense investigation in desperate need of predictive biomarkers to aid in surgical decision‐making. In the current study, we found that LDH was the strongest prognostic factor for OS both in the univariate and the multivariate analyses. Patients with elevated LDH had a nearly two‐fold higher risk for mortality (mOS, 27.6 months vs. 68.8 months). The 5‐year survival rate in the normal‐LDH and the high‐LDH groups was 53.7% and 22.5%, respectively (Figure 1). Although some scholars have investigated the utility of LDH as a serum biomarker in resectable and unresectable CRC, its usefulness has been limited by underpowered studies as well as its nonspecificity.^19^, ^20^, ^21^, ^22^, ^23^, ^24^, ^25^, ^26^ To the best of our knowledge, our study is the first to address the independent prognostic impact of preoperative LDH levels in curative‐intent CRLM resection.

Increased LDH is closely linked to hypoxia and angiogenesis in aggressive tumor phenotypes showing accelerated growth kinetics.^28^, ^29^, ^30^, ^31^, ^32^ The metabolism of fast‐growing cancer cells is shifted toward high glucose uptake and enhanced lactate production.^33^ In the TME, lactate promotes proinflammatory cytokines, such as TNF‐α, IL‐1, IL‐6, prostaglandins, and nuclear factor‐κB; enhances immune‐suppressive cells, such as myeloid‐derived suppressor cells (MDSCs) and dendritic cells (DCs); inhibits cytolytic cells, such as natural killer (NK) cells and cytotoxic T‐lymphocytes (CTLs); recruit tumor‐associated macrophages (TAMs) and promotes their conversion into immunosuppressive phenotype.^34^, ^35^, ^36^ Therefore, elevated LDH is a negative prognostic biomarker not only because of its key role in cancer metabolism, but also because it modulates the complex interplay between the TME and the host immune system, impacting the proliferation, invasion, and migration potential of malignant tumors.^37^

Interestingly, exploratory analysis unexpectedly showed that LDH levels strongly correlated with systemic inflammation indices, namely lymphocyte‐to‐monocyte ratio (LMR), lymphocyte‐to‐neutrophil ratio (LNR), and C‐reaction protein. In contrast, this correlation was not observed for CEA levels (data not shown). It has been reported that systemic inflammation leads to lymphocytopenia and increased the presence of TAMs, resulting in decreased cellular immunity.^38^, ^39^, ^40^, ^41^, ^42^ Meanwhile, growing evidence has shown that LDH could be a marker of diminished antitumor immunity, which inversely correlated with response to immune checkpoint blockade therapy.^43^ Moreover, the overexpression of hypoxia‐regulating factors, such as HIF‐1, Foxp3, and CCL‐28, might contribute to an immunosuppressive microenvironment by recruiting myeloid‐derived suppressor cells (MDSCs) and TAMs.^33^, ^44^ Thus, the mechanisms or pathways regulating LDH may intersect with hypoxia and antitumor immunity.^15^, ^32^, ^35^ LDH may serve as an alternative indicator of systemic inflammation and immunosuppression. LDH is recently emerging as an anticancer target.^45^, ^46^ Herein, we postulate that the perioperative use of nonsteroidal anti‐inflammatory drugs might decrease the recurrence risk of CRLM resection.^47^

It was reported that LDH could be the product of tumor necrosis due to hypoxia, which is a sign of a high tumor burden.^15^ In the present study, serum LDH levels did not show much relevance to most clinicopathologic parameters (such as primary tumor sidedness, T and N stage, *KRAS* status, pathology and differentiation, and disease‐free interval). Though elevated LDH was indeed associated with the maximum diameter of CRLM in our analysis, nevertheless, for patients with elevated LDH, 32.2% (30/93) of them virtually had the maximum diameter of CRLM below median value (2.5 cm) (Table 2). It was also worth noting that LDH levels were not associated with the number of CRLM. Perhaps more importantly, subgroup analysis showed that the prognostic value of LDH was independent of the number and size of CRLM. LDH also demonstrated even strong prognostic value among patients with extra‐hepatic metastases or with R1 surgical margin. Besides, LDH could distinguish the survival regardless of the Fong score (Figure 2).

The above findings suggested that the prognostic attribute of LDH in the current study might go beyond a simple indicator of heavier tumor burden. High LDH levels might denote aggressive biology in a way that is independent of traditional molecular and clinicopathologic features. LDH might be both a metabolic and an immune surveillance prognostic biomarker.

The prognostic scoring system proposed by Fong et al. (1990) has been widely used in clinical practice to stratify CRLM patients over time.^27^, ^48^, ^49^ Nevertheless, it has been questioned for rationality in current times.^50^, ^51^ The Fong score was originated from a single‐institution cohort, which might be influenced by local clinical practice patterns and biases. Therefore, it has not been successfully validated across different institutions,^52^, ^53^ especially in patients with long‐time follow‐up,^54^ or in the setting of neoadjuvant chemotherapy prior to hepatectomy.^55^, ^56^ Furthermore, in consideration of racial and genetic differences, data on Chinese populations were limited.

Though routine CEA test in CRC care is recommended globally, only 6.3% of patients in our data set had CEA >200 ng/ml, while a higher proportion of patients (16%) had elevated LDH. Consistent with recent studies,^9^, ^48^, ^49^, ^57^ we found that CEA had the insufficient statistical power to detect OS differences (*p* = 0.184). Notably, LDH could provide additional discriminatory ability on the basis of CEA and CA19‐9 levels. Specifically, among patients with CEA >5 ng/ml, median OS was distinguishable between patients with elevated and normal LDH levels (24.2 months vs. 60.6 months). For patients with CEA <5 ng/ml, elevated LDH still indicated worse OS (36.3 months vs. not reached). We observed an even more significant trend for CA19‐9 levels (Figure S2). Hence, LDH was a potential surrogate circulating tumor marker.

Importantly, LDH could classify 8.7% of the patients in the low‐risk group, 14.2% of the patients in the intermediate‐risk group, and 48.8% of patients in the high‐risk group with very distinct behaviors in the CRS model. Specifically, patients in the CRS 0–1 group who had LDH >ULN presented with significantly poor outcomes than patients who had LDH ≤ULN (OS 29.7 months vs. not reached, *p* = 0.005); conversely, patients in the CRS 4–5 group had LDH ≤ULN and presented with good outcomes than patients who had LDH >ULN (OS 44.8 months vs. 24.2 months, *p* < 0.001). Even for patients with CRS 2–3 group, patients who had LDH >ULN still demonstrated worse OS than patients with LDH ≤ULN (30.5 months vs. 60.2 months, *p* = 0.002). These data illustrated how the LDH levels overcame the CRS scoring system (Figure S3). Therefore, combing the Fong score with LDH, with a better prognostic discriminatory ability, outperformed the Fong score. Remarkably, both LDH‐CRS and mCRS identified a relatively higher proportion of patients in the high‐risk group (score 4–6) than CRS (13.2% vs. 12.0% vs. 8.5%). Thus, they could better define a portrait of the optimal candidate for CRLM resection with long‐term survival, as well as a picture of patients in whom direct hepatectomy may be ill‐advised and further neoadjuvant and adjuvant systemic therapy would be preferable.

We acknowledge that our analysis has some limitations due to its retrospective and observational nature. Some genetic parameters, including *RAS*, *BRAF*, microsatellite status, and postrelapse treatment, were not available in some data sets. It would be meaningful to combine LDH and specific mutations and molecular features of CRC in future. Recurrence time was not thoroughly recorded in cohort 2. Estimating of RFS was not stringently carried out at protocol‐specified intervals, though most physicians assessed the tumor status every 8–12 weeks. Less‐frequent assessment may bias in favor of a longer RFS time. Nevertheless, this factor is less likely to influence the primary OS outcome, which could genuinely reflect the clinical benefit of hepatectomy.^58^ Because the determination of the optimal cutoff value of LDH was beyond the scope of this study, we used the ULN to dichotomize this continuous variable, and the two participating centers adopted the same ULN of 250 U/ml. Finally, the enrollment dates of the two cohorts differed. Prospectively defined resectability criteria for CRLM were not established in the study protocol, the therapeutic decisions were made by a multidisciplinary team (MDT) in each medical center. However, since surgical interventions might outline a selection process per se, this would minimize the variations in patient selection between the two cohorts.

However, the above weaknesses had to be seen through the lens of clear strengths. The advantage of our study resided in the large sample size, the division in two independent cohorts, the long‐term follow‐up, and the heterogeneous cohort of unselected, real‐world patients. We also discovered that LDH might provide additional information on tumor metabolic and immune states. The accessibility and reproducibility of the noninvasive laboratory serum LDH test support its routine use in clinical practice. We expect future studies with prospective designs to validate our findings and a more explicit understanding of the molecular mechanisms of LDH in governing tumor biology.

## 5 CONCLUSION

Our study implicates preoperative LDH level as a reliable and independent laboratory biomarker to predict the outcome of curative‐intent surgery for CRLM. Integrating LDH into the established Fong scoring system can enhance the discrimination ability. Composite of LDH and Fong score is a potential stratification tool for CRLM resection. Prospective, international studies are needed to validate these results across diverse populations.

## 6 ETHICS STATEMENT

The authors declare that ethical approval has been acquired from the Research Ethics Committee of Sun Yat‐sen University Cancer Center and Sun Yat‐sen University Sixth Affiliated Hospital for this retrospective analysis. All patients were provided written consent for the use of their data at the time of hospitalization. All methods were carried out in accordance with Helsinki guidelines. No further ethical approval was required.

## 7 Consent for publication

All authors have read and approved the final version to be published and signed the author disclosure form.

## CONFLICT OF INTEREST STATEMENT

The authors declare that they have no conflicts of interest related to this research.

## Supporting information

Figure S1

Figure S2

Figure S3

Table S1

Table S2

Table S3

Table S4

Table S5

## Data Availability

The data sets used and/or analyzed during the current study are available from the corresponding author on reasonable request.
