# Supplementary figures and images for: The prognostic value of preoperative serum lactate dehydrogenase levels in patients underwent curative‐intent hepatectomy for colorectal liver metastases: A two‐center cohort study
Source: Cancer Med. 2021 Oct 12;10(22):8005–19. doi: 10.1002/cam4.4315 (PMC8607270; doi:10.1002/cam4.4315)

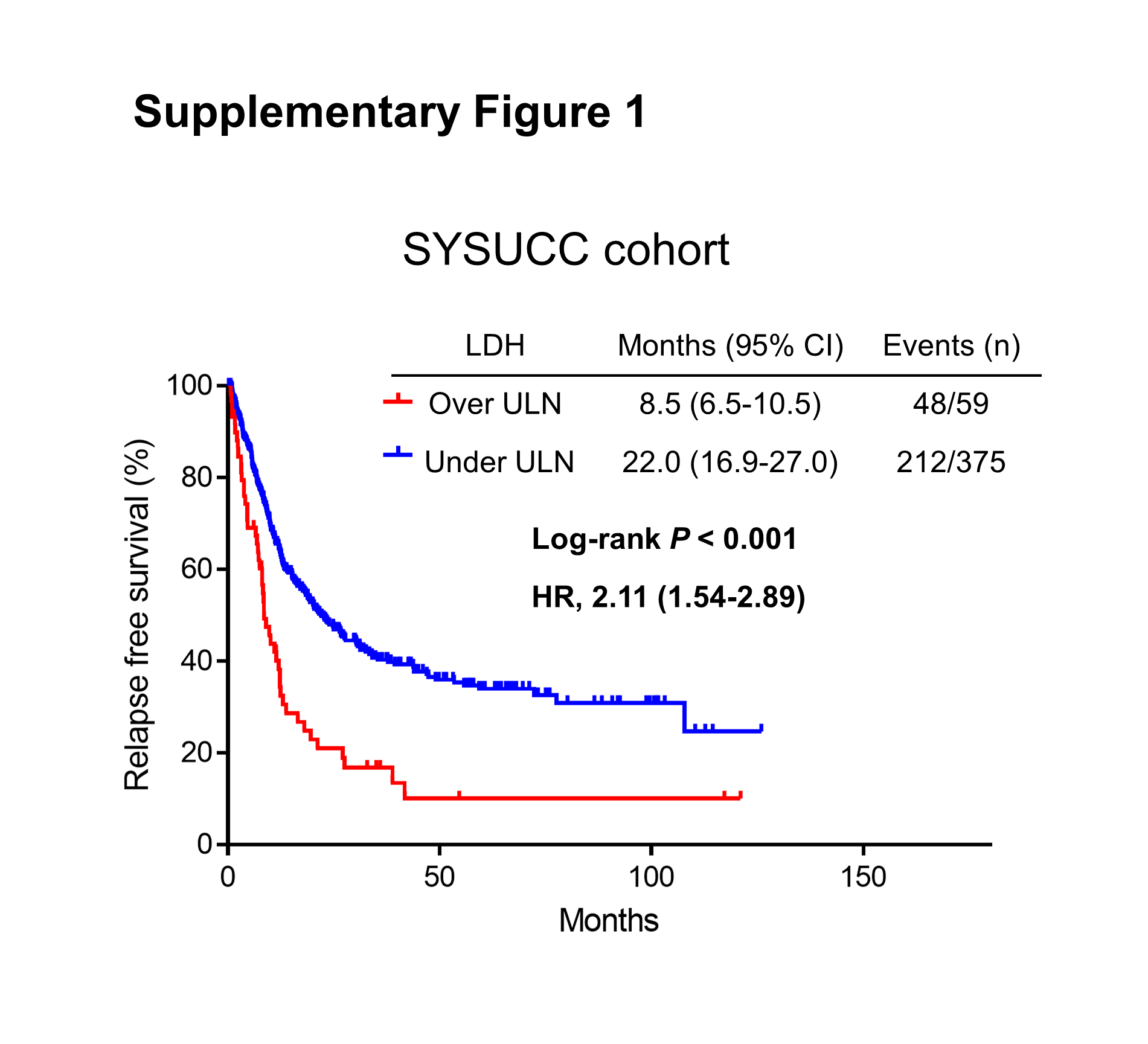

Supplement: Supplementary file 1 — Figure S1 [file CAM4-10-8005-s001.tif]

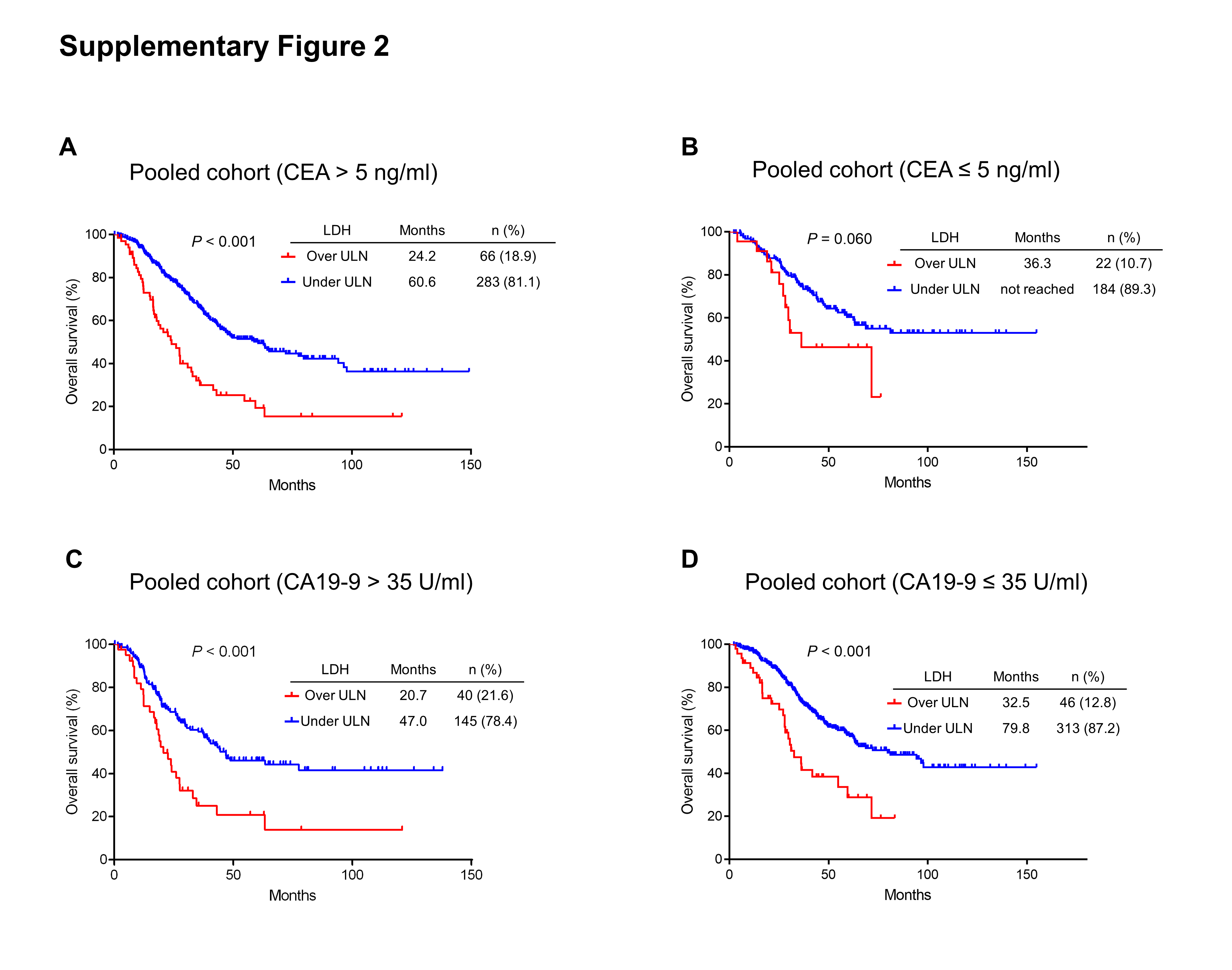

Supplement: Supplementary file 2 — Figure S2 [file CAM4-10-8005-s003.tif]

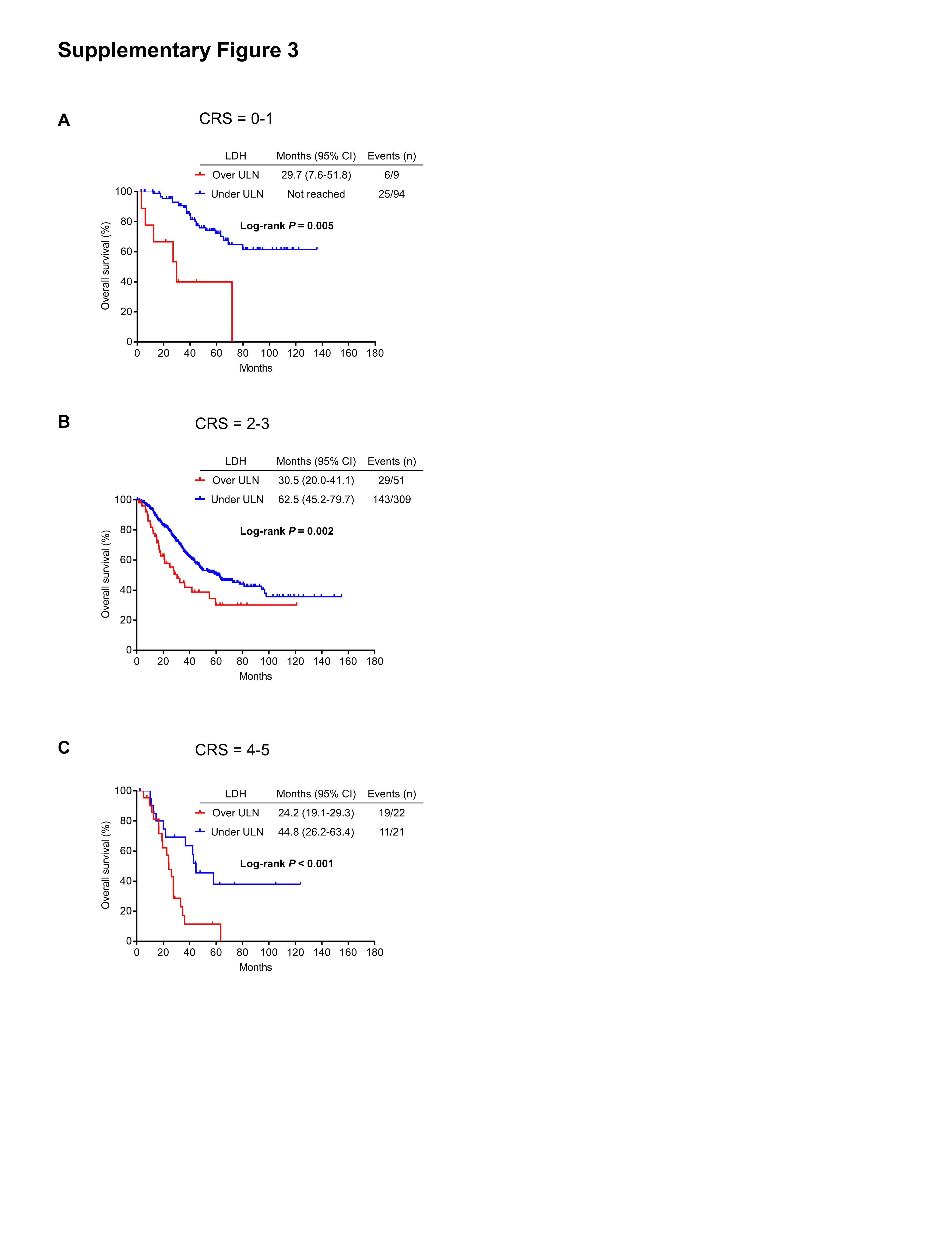

Supplement: Supplementary file 3 — Figure S3 [file CAM4-10-8005-s002.tif]
