# Supplementary material for: The prognostic value of preoperative serum lactate dehydrogenase levels in patients underwent curative‐intent hepatectomy for colorectal liver metastases: A two‐center cohort study
Source: Cancer Med. 2021 Oct 12;10(22):8005–19. doi: 10.1002/cam4.4315 (PMC8607270; doi:10.1002/cam4.4315)
Supplement: Supplementary file 4 — Table S1 [file CAM4-10-8005-s004.docx]

**Supplementary Table 1. Patient Clinicopathologic Characteristics between the two cohorts**

| **Variables** | **Cohort 1** | **Cohort 2** | ***P* value** |
| --- | --- | --- | --- |
|  | **n (%)** | **n (%)** |  |
| **Patient characteristics** |  |  |  |
| Age: median (range) | 57 (20-82) | 59 (24-80) | 0.507 |
| Gender |  |  | 1.000 |
| Male | 288 (66.4) | 97 (66.4) |  |
| Female | 146 (33.6) | 49 (33.6) |  |
| Preoperative CEA |  |  | 0.843 |
| > 200 ng/ml | 27 (6.6) | 8 (5.5) |  |
| ≤ 200 ng/ml | 383 (93.4) | 137 (94.5) |  |
| Preoperative LDH |  |  | 0.009* |
| Over ULN | 59 (13.6) | 34 (23.3) |  |
| Under ULN | 375 (86.4) | 112 (76.7) |  |
| **Survival outcome** |  |  |  |
| Median follow-up (month) | 65.5 (95% CI, 61.7-69.3) | 42.1 (95% CI, 36.7-47.5) | - |
| OS (month) | 58.9 (95% CI, 46.2-71.6) | 63.3 (95% CI, 61.3-67.8) | 0.444 |
| **Primary tumor characteristics** |  |  |  |
| Location ^a^ |  |  | 0.231 |
| Right-sided | 109 (32.3) | 30 (20.5) |  |
| Left-sided | 224 (67.3) | 116 (79.5) |  |
| Differentiation |  |  | 0.003* |
| Well/moderate | 331 (76.3) | 128 (87.7) |  |
| Poor | 103 (23.7) | 18 (12.3) |  |
| T stage |  |  | < .001* |
| Non-T4 | 258 (64.0) | 119 (83.2) |  |
| T4 | 145 (36.0) | 24 (16.8) |  |
| Lymph node metastases |  |  | 0.313 |
| Absent | 146 (40.6) | 51 (35.6) |  |
| Present | 213 (59.4) | 92 (64.4) |  |
| **CRLM characteristics** |  |  |  |
| Maximum diameter of CRLM |  |  | 0.578 |
| ≤ 5cm | 370 (86.4) | 120 (84.5) |  |
| > 5cm | 58 (13.6) | 22 (15.5) |  |
| Number of metastases |  |  | 0.264 |
| 1-2 | 296 (68.2) | 92 (63.0) |  |
| > 2 | 138 (31.8) | 54 (37.0) |  |
| Time of occurrence of CRLM |  |  | < .001* |
| Synchronous | 301 (69.4) | 139 (95.2) |  |
| Metachronous | 133 (30.6) | 7 (4.8) |  |
| R0 resection |  |  | 0.190 |
| Yes | 388 (89.4) | 116 (81.7) |  |
| No | 46 (10.6) | 26 (18.3) |  |

^a^ Colorectal cancer arising in or proximal to the splenic flexure was defined as right-sided; arising distal to the splenic flexure was defined as left-sided.

Abbreviations: LDH, lactate dehydrogenase; ULN, upper limit of normal; CRLM, colorectal liver metastases.

* indicates statistical significance.
