# Supplementary material for: The prognostic value of preoperative serum lactate dehydrogenase levels in patients underwent curative‐intent hepatectomy for colorectal liver metastases: A two‐center cohort study
Source: Cancer Med. 2021 Oct 12;10(22):8005–19. doi: 10.1002/cam4.4315 (PMC8607270; doi:10.1002/cam4.4315)
Supplement: Supplementary file 5 — Table S2 [file CAM4-10-8005-s007.docx]

**Supplementary Table 2.** Univariate and multivariate analyses for predictors of overall survival in cohort 1

| **Variables** | **Univariate analysis** | | | **Multivariate analysis** | | | |
| --- | --- | --- | --- | --- | --- | --- | --- |
|  | **HR (95% CI)** | ***P* value** | | | **HR (95% CI)** | ***P* value** | |
| Age | 1.25 (0.95-1.64) | | 0.105 | |  | |  |
| Gender (male) | 1.31 (0.98-1.75) | | 0.068 | | 1.58 (1.12-2.23) | | 0.009* |
| Primary tumor location ^a^ |  | |  | |  | |  |
| Right-sided vs. left-sided | 1.06 (0.78-1.45) | | 0.704 | |  | |  |
| Rectum vs. colon | 1.22 (0.93-1.59) | | 0.153 | |  | |  |
| Poor differentiation | 1.20 (0.89-1.63) | | 0.258 | |  | |  |
| T4 stage | 1.18 (0.89-1.57) | | 0.263 | |  | |  |
| Lymph node metastases | 1.77 (1.31-2.39) | | < .001 | | 1.89 (1.37-2.61) | | < .001* |
| Preoperative CEA levels  Preoperative CA19-9 levels | 1.06 (1.24-2.26)  1.49 (1.12-1.98) | | 0.001  0.006 | | 1.32 (0.93-1.87)  1.45 (1.32-1.87) | | 0.282  0.001* |
| Perioperative chemotherapy | 1.11 (0.76-1.61) | | 0.590 | |  | |  |
| Metachronous CRLM | 0.86 (0.65-1.15) | | 0.307 | |  | |  |
| Number of CRLM | 1.85 (1.40-2.44) | | < .001 | | 1.16 (1.09-1.24) | | < .001* |
| Maximum diameter of CRLM  R0 resection (Yes) | 2.17 (1.53-3.08)  0.32 (0.22-0.45) | | < .001  < .001 | | 1.16 (1.08-1.24)  0.62 (0.38-1.02) | | < .001*  0.058 |
| Extrahepatic disease (Yes) | 1.53 (1.03-2.27) | | 0.037 | | 1.97 (1.23-3.14) | | 0.005* |
| LDH levels (above ULN) | 2.41 (1.72-3.39) | | < .001 | | 1.77 (1.17-2.69) | | < .001* |

^a^ Colorectal cancer arising in or proximal to the splenic flexure was defined as right-sided; arising distal to the splenic flexure was defined as left-sided.

Abbreviations: HR, hazard ratio; CI, confidence interval; CRLM, colorectal liver metastases; ULN, upper limit of normal.

* indicates statistical significance.
