# Supplementary material for: The prognostic value of preoperative serum lactate dehydrogenase levels in patients underwent curative‐intent hepatectomy for colorectal liver metastases: A two‐center cohort study
Source: Cancer Med. 2021 Oct 12;10(22):8005–19. doi: 10.1002/cam4.4315 (PMC8607270; doi:10.1002/cam4.4315)
Supplement: Supplementary file 6 — Table S3 [file CAM4-10-8005-s008.docx]

**Supplementary Table 3.** Univariate and multivariate analyses for predictors of overall survival in cohort 2

| **Variables** | **Univariate analysis** | | | **Multivariate analysis** | | | |
| --- | --- | --- | --- | --- | --- | --- | --- |
|  | **HR (95% CI)** | ***P* value** | | | **HR (95% CI)** | ***P* value** | |
| Age | 1.29 (0.73-2.28) | | 0.379 | |  | |  |
| Gender (male) | 1.28 (0.72-2.28) | | 0.403 | |  | |  |
| Primary tumor location |  | |  | |  | |  |
| Right-sided vs. left-sided ^a^ | 1.79 (0.95-3.36) | | 0.073 | | 2.44 (1.23-4.83) | | 0.011* |
| Rectum vs. colon | 0.68 (0.39-1.19) | | 0.173 | |  | |  |
| Poor differentiation | 0.57 (0.28-1.18) | | 0.129 | |  | |  |
| T4 stage | 2.26 (1.17-4.37) | | 0.015 | | 2.14 (1.01-4.54) | | 0.047* |
| Lymph node metastases | 1.96 (1.03-3.76) | | 0.042 | | 2.29 (1.16-4.53) | | 0.017* |
| Preoperative CEA levels  Preoperative CA19-9 levels  R0 resection | 1.84 (0.73-4.65)  1.98 (1.36-3.32)  0.83 (0.39-1.77) | | 0.196  0.068  0.628 | | 1.34 (0.73-2.38) | | 0.478 |
| Metachronous CRLM | 0.42 (0.17-1.08) | | 0.071 | | 0.22 (0.73-0.66) | | 0.007* |
| Maximum diameter of CRLM | 1.39 (0.78-2.47) | | 0.267 | |  | |  |
| Number of CRLM | 1.12 (0.98-1.28) | | 0.104 | |  | |  |
| LDH level (above ULN) | 3.16 (1.75-5.70) | | < .001 | | 3.71 (1.75-7.89) | | 0.001* |

^a^ Colorectal cancer arising in or proximal to the splenic flexure was defined as right-sided; arising distal to the splenic flexure was defined as left-sided.

Abbreviations: HR, hazard ratio; CI, confidence interval; CRLM, colorectal liver metastases; ULN, upper limit of normal.

* indicates statistical significance.
