# Supplementary material for: The prognostic value of preoperative serum lactate dehydrogenase levels in patients underwent curative‐intent hepatectomy for colorectal liver metastases: A two‐center cohort study
Source: Cancer Med. 2021 Oct 12;10(22):8005–19. doi: 10.1002/cam4.4315 (PMC8607270; doi:10.1002/cam4.4315)
Supplement: Supplementary file 7 — Table S4 [file CAM4-10-8005-s006.docx]

**Supplementary Table 4.** Univariate and multivariate analyses for predictors of relapse-free survival in cohort 1

| **Variables** | **Univariate analysis** | | | **Multivariate analysis** | | | |
| --- | --- | --- | --- | --- | --- | --- | --- |
|  | **HR (95% CI)** | ***P* value** | | | **HR (95% CI)** | ***P* value** | |
| Age | 1.00 (0.78-1.29) | | 0.989 | |  | |  |
| Gender (male) | 1.16 (0.89-1.50) | | 0.270 | |  | |  |
| Primary tumor location |  | |  | |  | |  |
| Right-sided vs. left-sided ^a^ | 1.03 (0.757-1.407) | | 0.840 | |  | |  |
| Rectum vs. colon | 0.95(0.716-1.259) | | 0.719 | |  | |  |
| Poor differentiation | 1.10 (0.83-1.46) | | 0.494 | |  | |  |
| T4 stage | 1.11 (0.86-1.45) | | 0.420 | |  | |  |
| Lymph node metastases | 1.73 (1.32-2.28) | | < .001 | | 1.76 (1.32-2.35) | | < .001* |
| Perioperative chemotherapy | 1.43 (1.00-2.05) | | 0.048 | | 1.35 (0.93-1.95) | | 0.113 |
| Preoperative CEA levels  Preoperative CA19-9 levels | 1.18 (0.72-1.94)  1.29 (1.09-1.68) | | 0.508  0.012 | | 1.19 (0.83-1.71) | | 0.235 |
| Metachronous CRLM | 0.98 (0.75-1.28) | | 0.874 | |  | |  |
| Number of CRLM | 1.20 (1.15-1.25) | | < .001 | | 1.17 (1.10-1.23) | | < .001* |
| Maximum diameter of CRLM | 1.16 (1.10-1.23) | | < .001 | | 1.12 (1.05-1.20) | | 0.001* |
| Extrahepatic disease  R0 resection (Yes) | 1.82 (1.29-2.57)  0.42 (0.30-0.59) | | 0.001  < .001 | | 1.44 (0.95-2.16)  0.97 (0.61-1.54) | | 0.084  0.887 |
| LDH level (above ULN) | 2.11 (1.54-2.89) | | < .001 | | 1.53 (1.01-2.03) | | 0.042* |

^a^ Colorectal cancer arising in or proximal to the splenic flexure was defined as right-sided; arising distal to the splenic flexure was defined as left-sided.

Abbreviations: HR, hazard ratio; CI, confidence interval; CRLM, colorectal liver metastases; ULN, upper limit of normal.

* indicates statistical significance.
