# Supplementary material for: The prognostic value of preoperative serum lactate dehydrogenase levels in patients underwent curative‐intent hepatectomy for colorectal liver metastases: A two‐center cohort study
Source: Cancer Med. 2021 Oct 12;10(22):8005–19. doi: 10.1002/cam4.4315 (PMC8607270; doi:10.1002/cam4.4315)
Supplement: Supplementary file 8 — Table S5 [file CAM4-10-8005-s005.docx]

**Supplementary Table 5. Univariate and multivariate analyses for predictors of overall survival in patients with available *KRAS* mutation status in the pooled cohort**

| **Variables** | **Univariate analysis** | | | **Multivariate analysis** | | | |
| --- | --- | --- | --- | --- | --- | --- | --- |
|  | **HR (95% CI)** | ***P* value** | | | **HR (95% CI)** | ***P* value** | |
| Age | 1.01 (0.99-1.03) | | 0.190 | |  | |  |
| Gender (male) | 1.22 (0.71-2.09) | | 0.471 | |  | |  |
| *KRAS* mutation | 1.64 (1.02-2.64) | | 0.041 | | 1.62 (0.35-1.14) | | 0.062 |
| Primary tumor location ^a^ |  | |  | |  | |  |
| (Right-sided vs. left-sided) | 1.42 (0.82-2.45) | | 0.215 | |  | |  |
| Poor differentiation | 1.25 (0.71-2.23) | | 0.441 | |  | |  |
| T4 stage | 1.10 (0.66-1.82) | | 0.722 | |  | |  |
| Lymph node metastases | 1.10 (0.68-1.79) | | 0.698 | |  | |  |
| Preoperative CEA levels | 1.00 (0.99-1.00) | | 0.843 | |  | |  |
| Metachronous CRLM | 0.61 (0.35-1.07) | | 0.084 | | 0.63 (0.98-1.14) | | 0.125 |
| Number of CRLM | 1.23 (1.13-1.34) | | < .001 | | 1.25 (1.13-1.38) | | < .001* |
| Maximum diameter of CRLM | 1.22 (1.07-1.39) | | 0.003 | | 1.17 (1.00-1.37) | | 0.050 |
| LDH levels | 1.31 (0.73-2.35) | | 0.065 | | 1.07 (0.56-2.02) | | 0.798 |

^a^ Colorectal cancer arising in or proximal to the splenic flexure was defined as right-sided; arising distal to the splenic flexure was defined as left-sided.

Abbreviations: HR, hazard ratio; CI, confidence interval; CRLM, colorectal liver metastases.

* indicates statistical significance.
